# Internet Search Activity for Intentional Self-Harm Forums After a High-Profile News Publication: Interrupted Time Series Analysis

**DOI:** 10.2196/52354

**Published:** 2024-10-15

**Authors:** Nora Clancy Kelsall, Catherine Gimbrone, Mark Olfson, Madelyn Gould, Jeffrey Shaman, Katherine Keyes

**Affiliations:** 1 Department of Epidemiology Columbia University New York, NY United States; 2 Department of Psychiatry Columbia University New York, NY United States; 3 Department of Environmental Health Sciences Columbia University New York, NY United States; 4 Columbia Climate School Columbia University New York, NY United States

**Keywords:** suicide risk, suicide, journalism, media, self-harm, Google Trends, websites, mental health, depression, quality of life, harmful information

## Abstract

Searches for “pro-suicide” websites in the United States peaked during the week a high-profile news story was published and remained elevated for 6 months afterward, highlighting the need to avoid mentioning specific sources of explicit suicide instructions in media publications.

## Introduction

Suicide has substantially increased in the United States, especially among young people [1]. There is evidence that exposure to explicit content about suicide, most notably after reports of high-profile suicide deaths, increases population rates of suicide [2,3]. Many studies have focused on news or television media, whereas individuals seek information and social support on social media and other online resources [4]. “Pro-suicide” websites are those that promote or facilitate suicide, including advocating for an individual’s “right” to end their own life, creating communities of individuals who desire to end their own lives, as well as sharing explicit information on means of killing oneself [5,6]. These sites provide largely unmoderated forums for discussion of suicide, which may encourage, promote, or facilitate suicide. These sites are accessible and commonly used by individuals experiencing suicidal thoughts and otherwise vulnerable individuals [7]. Therefore, understanding trends in accessing these sites could inform preventive interventions and media practices when discussing such sites.

In 2021, a national news source published a story about pro-suicide websites and named one such site. We examined whether searches for the named site and similar sites increased following its publication.

## Methods

National weekly Google data covering 6 months before and after the publication were drawn from the Google Health Trends API [8]. We queried search terms, including names of pro-suicide websites (available upon request). Queries randomly return 10% to 15% of total Google searches in the United States for given time periods as well as the probability of a short search session for the given terms per 10 million searches. We requested 10 datasets, which we averaged to estimate weekly search levels.

We first graphed the weekly time trend. To estimate whether searches increased during the week of the news publication, we used search activity from the previous 6 months to forecast expected search levels using an autoregressive integrated moving average (ARIMA) model. We then compared the expected search levels to the observed levels for that week.

An interrupted time series model was used to estimate the effects of the publication on the intercept and slope time trends 6 months after publication, removing the week of the publication. The model was defined as:

*E*(*Y*) = β_1_ + β_2_ * *T*_1_ + β_3_ * *P* + β_4_ * *T*_2_      **(1)**

with *T*_1_ as time since the beginning of the measurement period, *T*_2_ as time since publication, and *P* as a binary indicator of before/after article publication.

## Results

**Figure 1:**
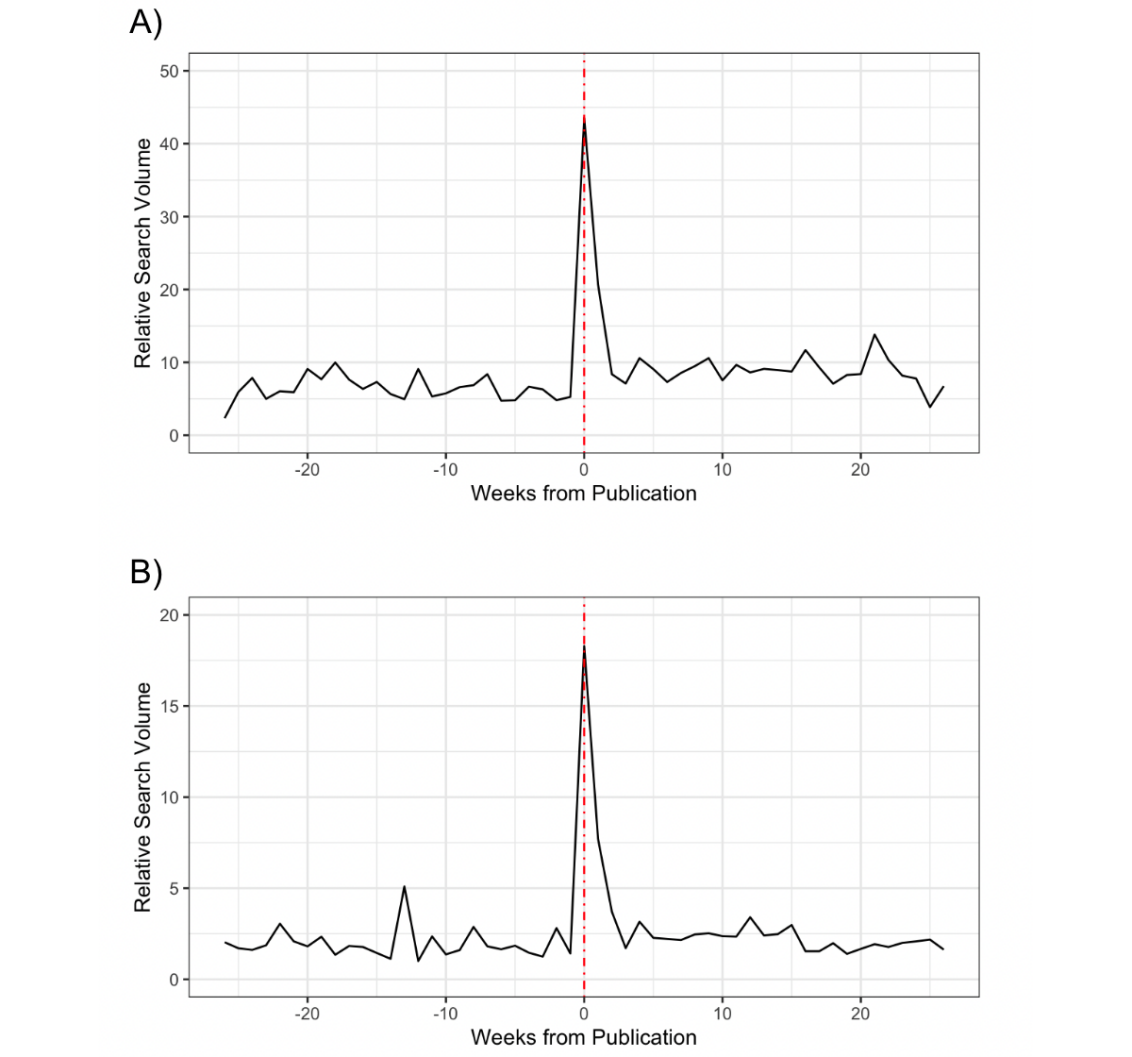
Time trends for searches related to (A) the website named in the news article and (B) pro-suicide websites.

Figure 1 shows the average weekly relative search volume for the abovementioned website (panel A) and searches for a broader range of pro-suicide websites (panel B), indexed by time of the news story. In the week of the news story’s publication, searches for pro-suicide websites and the mentioned website were 564% (95% CI 366%-1108%) and 842% (95% CI 393%-2979%) higher, respectively, than what would be expected based on the prior 6 months.

After excluding the week of publication, the interrupted time series model results showed that there was an intercept effect of searches for suicide websites increasing 80.2% (95% CI 41.0%-119%, *P*<.001; intercept B=5.26, 95% CI 2.69-7.83). Searches for the mentioned website increased by 90.2% (95% CI 34.0%-146%, *P*=.003; intercept B=1.75, 95% CI 0.66-2.84) compared to the mean of the prior 6 months, with no change in difference over time.

## Discussion

Searches for pro-suicide websites in the United States peaked in the week of the publication of a high-profile news story and remained higher than prepublication times in the 6 months that followed. While we do not know the level of suicide risk for those accessing these sites and have no means of attributing increased searches to suicidal behavior, these results indicate that news attention to pro-suicide forums may increase the number of individuals seeking access.

Journalism around suicide requires balancing public interest with well-documented contagion and trigger phenomena [3]. For pro-suicide websites, there is a clear public interest in informing parents and providers about potentially harmful information. Yet publicity about pro-suicide websites has the potential to increase awareness among those vulnerable to its content. Providing accurate information and safe online spaces to discuss suicide may counteract more dangerous sources on the internet [9]. Further, for all terms searched in our study, Google lists the national 988 Suicide and Crisis Lifeline as the first result, which may mitigate potential harms. Nevertheless, we caution against mentioning specific sources of explicit instructions for suicide in media publications. Although internet use varies by geographical region and age group, the use of national Google Trends data does not allow for differentiating search volumes by user demographics or intent.

## Abbreviations

ARIMA: autoregressive integrated moving average

## Data Availability

The data used in these analyses are not publicly available in order to limit dissemination of pro-suicide website names but are available from the corresponding author upon reasonable request.
